# EHEALTH PROGRAM BASED ON THE INFORMATION-MOTIVATION-BEHAVIORAL SKILLS MODEL FOR THE ELDERLY: A PILOT STUDY

**DOI:** 10.1093/geroni/igz038.1088

**Published:** 2019-11-08

**Authors:** Eunjin Yang, Sun Ju Chang, Kyoung-eun Lee, Hyunju Ryu

**Affiliations:** 1 College of Nursing, Seoul National University, Seoul, Korea, Republic of; 2 College of Nursing & Research Institute of Nursing Science, Seoul National University, Seoul, Korea, Republic of

## Abstract

Although the internet is helpful and considered a preferred channel to get health information, some vulnerable populations such as the elderly have a digital divide. The purpose of this study was to test the development and intervention of the eHealth program based on the Information-Motivation-Behavioral Skills (IMB) model for improving internet health information seeking, understanding and utilization behaviors in the elderly. This study was a single group pretest-posttest design, and the eleven elderly aged 67-87 (mean74.6, SD 6.9) participated in 5 session program at a senior welfare center from 25 January to 22 February 2019. Each theory-based constructs of IMB model such as computer/web knowledge (p<.01), attitude toward the internet health information usage (p<.01), and eHealth literacy (p<.01) was significantly improved at posttest than pretest. More than half of the participants (6 of 11) gave up on searching internet health information on pretest; however, all of the participants searched the internet health information accurately on the posttest. Regarding health information understanding, a significant difference was found (p=.03), and participants reported positive behavioral change after the program (6.54 ± 2.42). This pilot study indicated that the theory-based eHealth program might be an effective way to decrease a digital divide for the elderly. Therefore, the preliminary findings show promise for the use of the IMB model-based eHealth program as an intervention to improve internet health information seeking, understanding, and utilization behaviors in the elderly.

